# Efficacy and safety of traditional Chinese medicine external washing in the treatment of postoperative wound of diabetes complicated with anal fistula: Study protocol of a randomized, double-blind, placebo-controlled, multi-center clinical trial

**DOI:** 10.3389/fphar.2022.938270

**Published:** 2022-12-14

**Authors:** Jian Kang, Ya Liu, Sihan Peng, Xiao Tang, Lu Liu, Ziyan Xie, Yuchi He, Xiyu Zhang

**Affiliations:** ^1^ Department of Anorectal, Hospital of Chengdu University of Traditional Chinese Medicine, Chengdu, China; ^2^ Hospital of Chengdu University of Traditional Chinese Medicine, TCM Regulating Metabolic Diseases Key Laboratory of Sichuan Province, Chengdu, China; ^3^ School of Clinical Medicine, Chengdu University of Traditional Chinese Medicine, Chengdu, China

**Keywords:** postoperative diabetes with anal fistula, traditional Chinese medicine, external washing, randomized controlled trial, Jiedu Shengji decoction

## Abstract

**Introduction:** Anal fistula is one of the commonest ailments seen by anorectal surgeons as surgery is currently the preferred treatment for it. Diabetes mellitus is a risk factor that can lead to slow wound healing after anal fistula surgery. Because of the large postoperative wound surface of anal fistula, patients with diabetes can have an increased probability of wound infection, which makes it hard to heal. There is an extensive clinical experience for wound healing in traditional Chinese medicine (TCM). The Jiedu Shengji decoction (JSD) is a widely used external washing decoction in clinical practice. However, the current evidence on it is still insufficient. Therefore, we report this carefully designed clinical trial to assess the efficacy and safety of JSD in the treatment of postoperative wounds in diabetic patients with anal fistula.

**Methods and analysis:** This study was designed to be a randomized, double-blind, placebo-controlled, multi-center clinical trial. There were 60 eligible participants who were randomized at a 1:1 ratio to the intervention and placebo groups. Both groups received the same standard treatment. The intervention group was given external washing decoction of TCM (JSD), while the placebo group was given the placebo made of excipients and flavoring agents. The main outcome measures include wound healing, distribution of wound pathogens, levels of inflammatory mediators, and blood glucose. The secondary outcome measures included lipids, the quality of the life evaluation scale (Short-Form Health Survey 36). Assessments were performed before the start of the study, at 1st, 2nd, 3rd, and 4th weeks after the intervention, and at 8th, 12th, and 16th follow-up weeks.

**Discussion:** The clinical study we proposed will be the first randomized, double-blind, placebo-controlled, multi-center clinical trial study to assess the efficacy and safety of TCM external washing (JSD) in the treatment of postoperative wounds in diabetic patients with anal fistula.

**Ethics and dissemination:** The Medical Ethics Committee of Hospital of Chengdu University of Traditional Chinese Medicine has reviewed this study protocol and gave its approval and consent on 17 March, 2022 (Ethical Review Number: 2022KL-018).

## 1 Introduction

Anal fistula is one of the commonest ailments seen by anorectal surgeons. The prevalence of fistula-in-ano is 12.3 cases per 100,000 population in men and 5.6 cases per 100,000 population in women (Mei et al., 2019). Patients with anal fistula usually present with a recurrent abscess or a draining fistula with various severities of symptoms and require surgical interventions (Pigot, 2015; Lu et al., 2019). At present, the preferred treatment on anal fistula is surgery, which is a way to completely cure it, with an unequivocal efficacy and low recurrence rate (Shi et al., 2021). However, the large postoperative wound surface, which can lead to a high probability of wound infection and long healing time, is the reason which causes physical and psychological fear in patients (Kochhar et al., 2014; Farag et al., 2019).

According to the 10th edition of the International Diabetes Federation Diabetes Atlas, it is estimated that the global diabetes prevalence is 10.5% (536.6 million people) in population aged from 20 to 79 in 2021, and this number will rise to 12.2% (783.2 million) in 2045 (Sun et al., 2022). Perianorectal abscess can easily happen on patients with diabetes due to their reduced skin resistance, which might develop into anal fistula. In addition, the high blood glucose level of diabetic patients can provide a favorable nutritional environment for bacteria to grow, which can easily cause postoperative wound infection or even necrosis to slow down wound healing. Studies have shown that diabetes is a risk factor leading to slow wound healing after anal fistula surgery (Wang et al., 2014; Mei et al., 2019; Wang et al., 2021).

Traditional Chinese medicine can exert great clinical effects on wound healing (Mosavat et al., 2015; Yan et al., 2020). In particular, TCM external washing has a long history, extensive experience, and positive efficacy in promoting wound healing after anal fistula surgery and has been commonly used for postoperative treatment in China. In traditional Chinese medicine, it is believed that dampness, heat, and static blood are the pathological characteristics of postoperative wounds in diabetic patients with anal fistula.

The Jiedu Shengji decoction (JSD) is a widely used external washing decoction in clinical practice, which has the functions of removing toxins for detumescence, discharging pus, and promoting granulation. The main botanical herbs in JSD are Zi Cao (*Arnebia euchroma* (Royle) Johnst.), Dang Gui (*Angelica sinensis* (Oliv.) Diels.), Ru Xiang (*Boswellia carterii* Birdw.), Mo Yao (*Commiphora myrrha* (T.Nees) Engl.), Xue Jie (*Daemonorops draco* (Willd.) Blume), Bing Pian (*Cinnamomum camphora* (L.) J.Presl), Bai Zhi (*Angelica dahurica* (Hoffm.) Benth. Et] Hook. f. ex Franch. et Sav), and Er Cha (*Acacia catechu* (L. F.) Willd). Preliminary studies have found that JSD can significantly reduce wound edema after anal fistula surgery, reduce nerve sensitivity, ease the pain in patients, and promote wound healing. However, this study was limited by its single-center and non-double-blind design. Therefore, a multi-center, double-blind, randomized, controlled clinical trial is needed to further determine the efficacy and safety of JSD in the treatment of postoperative wounds in diabetic patients with anal fistula. We proposed that patients treated with JSD will have more positive clinical outcomes than those with placebos.

## 2 Methods/design

### 2.1 Trial design

**FIGURE 1:**
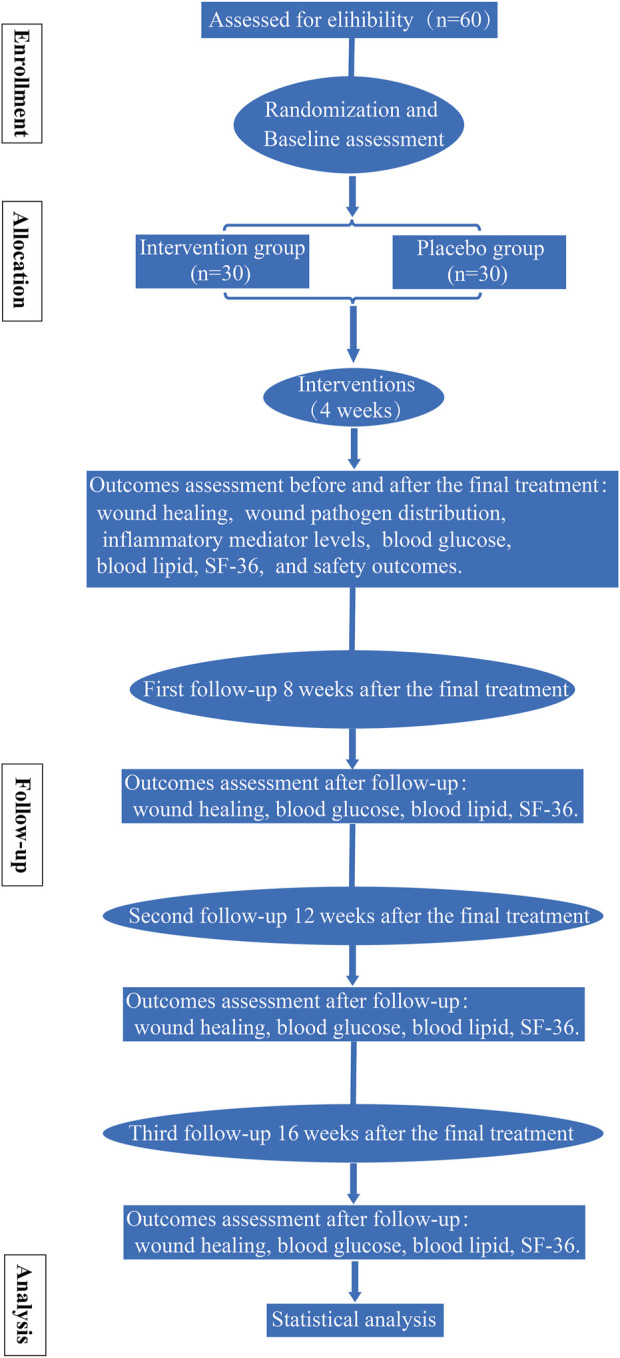
Study process: flowchart of the study procedure.

This study incorporates a randomized, double-blind, placebo-controlled, multi-center clinical trial and was developed according to the Standard Protocol Items: Recommendations for Interventional Trials (SPIRIT) statement (the SPIRIT checklist is shown in Supplementary Material S1). Every eligible participant was assigned either to the intervention group or the placebo group randomly. All patients received standard treatment. The detailed workflow is shown in Figure 1. The trial is supported by the science and technology planning project of Sichuan Province [grant no. 2021YFS0275]. The funders have no vote in the study design, data collection and analysis, manuscript preparation, or decision to publish.

### 2.2 Study population

Patients were recruited from three hospitals: the Affiliated Hospital of Chengdu University of Traditional Chinese Medicine, Yanjiang District Hospital of Traditional Chinese Medicine in Ziyang City, and Chengdu Anorectal Specialized Hospital.

### 2.3 Recruitment of participants

Three members (ZX, YL, and YHP) were involved in recruiting participants, and the other two members (SP and LL) were responsible for assigning participants to groups. Patients who met the study criteria and volunteered to participate in the study were asked to sign a written informed consent form (Supplementary Material S2) with a clear understanding of the purpose, procedures, and all potential risks associated with the study. Participants’ personal information was kept confidential, and only authorized researchers will have access to them. All paper forms pertaining to this study were kept in a locked and secure office. Participants may obtain dataset from respective authors upon reasonable request.

### 2.4 Patient and public involvement

Patients or the public were not involved in the design, conduct, reporting, or dissemination plans of our research.

### 2.5 Inclusion criteria

1. Confirmed diabetes mellitus (Alberti and Zimmet, 1998) with postoperative anal fistula.

The diagnostic criteria for anal fistula refer to the guidelines for Clinical Diagnosis and Treatment of Anal Fistula (2006 Edition) jointly formulated by the Colorectal and Anal Surgery Group of Chinese Society of Surgery, Anorectal Branch of Chinese Society of Traditional Chinese Medicine, and Specialized Committee of Colorectal and Anal Diseases, Chinese Association of Modern Medicine.

2. Aged 18 to 75.
3. 4cm^2^ ≤ postoperative wound area<30 cm^2^.
4. TCM syndrome differentiation is dampness–heat blood stasis syndrome, referring to the People’s Republic of China Industry Standard for Traditional Chinese Medicine Diagnosis and Efficacy Criteria for Anal Leakage Syndrome.
5. No previous surgery for anorectal disease and no abnormal anal morphology or function.
6. Voluntary participation and signed informed consent.

### 2.6 Exclusion criteria

1. Patients with severe diseases in important organs, including abnormal liver function, renal failure, heart failure, stroke, and malignant tumors.
2. Participated in other clinical trials within 3 months before enrollment.
3. Allergic to known drugs or experimental drugs.
4. Pregnant and lactating women.
5. Obvious mental disorders.

### 2.7 Handling of withdrawal and data management

Participants can withdraw from the study at any time. If any participant stops using study medication or takes other medications that may affect the results during the study, the participant will not be a part of the study anymore. Participants failed to follow up and ones withdrawn from the study were recorded and reported. Incomplete data will be cleaned up if less than 5–10% of participants withdraw from the study. Missing data will be processed through data imputation, intention to treat analysis, and sensitivity analysis if greater than 10% participants withdraw from the study.

### 2.8 Interventions

All participants went through an interview to know more information about this study. After obtaining informed consent and completing baseline assessments, eligible participants were randomized to the intervention and placebo groups. Participants were not allowed to take any other Chinese herbal decoction or Chinese patent medicine during the study.

**FIGURE 2:**
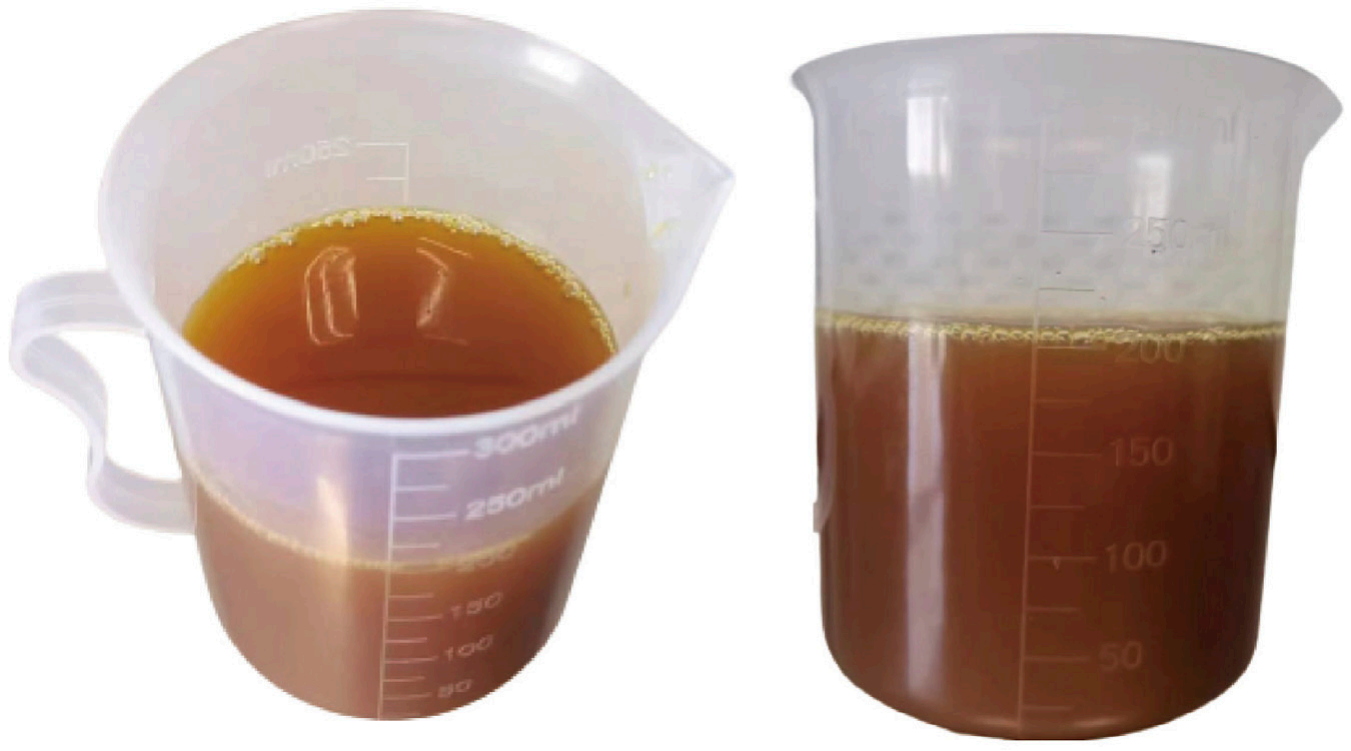
Jiedu Shengji decoction (JSD).

Both groups received standard treatments, including health knowledge education regarding diabetes and anal fistula, formulations of recipes, and taking hypoglycemic, antihypertensive, hypolipidemic, and anti-infective drugs. A personalized treatment plan was formulated after consultation with an endocrinologist, and the plan was evaluated and adjusted throughout the process. The wound surface was routinely cleaned with povidone-iodine after daily defecation. The intervention group was given TCM external washing decoction, JSD (Figure 2), and the information of its constituent botanical drugs is shown in Table 1. The JSD decoction was prepared by the Department of Pharmacy, Hospital of Chengdu University of Traditional Chinese Medicine. The specific steps are as follows: (1) Bai Zhi, Zi Cao, and Dang Gui are added to 500 ml of water and soaked for 30 min, and the mixture of water and herbs is transferred to the decoction machine, boiled at high heat to 100°C under standard atmospheric pressure (the boiling time to 100°C should not be more than 5 min), turned to low heat and maintained the temperature constant at 85°C–95°C, then continued to decoct for 30 min, obtained 200 ml of the botanical drug liquid was filtered, and set aside. (2) A measure of 500 ml of water is added again, boiled to 100°C on high heat (boiling time to 100°C should not be more than 5min), turned down the heat and maintained the temperature constant at 85°C–95°C, and decocted for 45 min. (3) The liquid from the previous two steps are mixed to obtain a total of 400 ml of the liquid (liquid 1). (4) Grind Ru Xiang, Mo Yao, and Er Cha, in advance, are filtered through a 2-mm sieve, 400 ml of water is added, boiled in high heat to 100°C (boiling time to 100°C should not be longer than 5 min), turned down the heat and maintained the temperature constant at 85°C–95°C, added pre-ground Xue Jie and Bing Pian (filtered through 2 mm sieve) at the 14th min, continued to boil for 2 min to get 200 ml of the liquid (liquid 2). (5) Liquid 1 and liquid 2 are mixed thoroughly to get 600 ml of liquid. (6) After waiting for its temperature to 35°C–40°C, 200-ml herbal medicine bags are plasticized. The decoction was produced in the same batch as far as possible and quality-checked by the manufacturer. To ensure that the decoction is not contaminated, the decoction vessels and filtration and dispensing equipment were completely cleaned in advance to make sure there were no residuals.

**TABLE 1:** Components of JSD.

Name of the drug	Use part	Manufacturer	Dose (g)	Method of use	Dosage form, dosage, frequency, and duration of treatment	Registration or not (Y/N)	Quality control report? (Y/N)	Storage condition
Zi Cao [*Arnebia euchroma* (Royle) Johnst.]	Whole plants	Sichuan Chinese Herb Preparation Co., Ltd., Sichuan, China	15	Washing and sitz bath for 30 min	Liquid, 200 ml, once a day, last 4 weeks	Y-registered in relevant administration	Y-prepared according to the manufacturer	The drugs are sealed and stored in a Chinese medicine warehouse at 0–30°C and 45–75% humidity
Dang Gui [*Angelica sinensis* (Oliv.) Diels.]	Roots	Sichuan Chinese Herb Preparation Co., Ltd., Sichuan, China	20	Washing and sitz bath for 30 min	Liquid, 200ml, once a day, last 4 weeks	Y-registered in relevant administration	Y-prepared according to the manufacturer	The drugs are sealed and stored in a Chinese medicine warehouse at 0–30°C and 45–75% humidity
Ru Xiang [*Boswellia carterii* birdw.]	Resin	Sichuan Chinese Herb Preparation Co., Ltd., Sichuan, China	15	Washing and sitz bath for 30 min	Liquid, 200ml, once a day, last 4 weeks	Y-registered in relevant administration	Y-prepared according to the manufacturer	The drugs are sealed and stored in a Chinese medicine warehouse at 0–30°C and 45–75% humidity
Mo Yao [*Commiphora myrrha* (T.Nees) Engl.]	Resin	Sichuan Chinese Herb Preparation Co., Ltd., Sichuan, China	15	Washing and sitz bath for 30 min	Liquid, 200 ml, once a day, last 4 weeks	Y-registered in relevant administration	Y-prepared according to the manufacturer	The drugs are sealed and stored in a Chinese medicine warehouse at 0–30°C and 45–75% humidity
Xue Jie [*Daemonorops draco* (Willd.) Blume]	Resin	Sichuan Chinese Herb Preparation Co., Ltd., Sichuan, China	15	Washing and sitz bath for 30 min	Liquid, 200ml, once a day, last 4 weeks	Y-registered in relevant administration	Y-prepared according to the manufacturer	The drugs are sealed and stored in a Chinese medicine warehouse at 0–30°C and 45–75% humidity
Bing Pian [*Cinnamomum camphora* (L.) J.Presl]	Extract	Sichuan Chinese Herb Preparation Co., Ltd., Sichuan, China	5	Washing and sitz bath for 30 min	Liquid, 200 ml, once a day, last 4 weeks	Y-registered in relevant administration	Y-prepared according to the manufacturer	The drugs are sealed and stored in a Chinese medicine warehouse at 0–30°C and 45–75% humidity
Bai Zhi [*Angelica dahurica* (Hoffm.) Benth. Et] Hook. f. ex Franch. et Sav]	Roots	Sichuan Chinese Herb Preparation Co., Ltd., Sichuan, China	20	Washing and sitz bath for 30 min	Liquid, 200ml, once a day, last 4 weeks	Y-registered in relevant administration	Y-prepared according to the manufacturer	The drugs are sealed and stored in a Chinese medicine warehouse at 0–30°C and 45–75% humidity
Ercha [*Acacia catechu* (L. F.) Willd]	Dry extract of skin, branch, and stem	Sichuan Chinese Herb Preparation Co., Ltd., Sichuan, China	20	Washing and sitz bath for 30 min	Liquid, 200ml, once a day, last 4 weeks	Y-registered in relevant administration	Y-prepared according to the manufacturer	The drugs are sealed and stored in a Chinese medicine warehouse at 0–30°C and 45–75% humidity

The placebo group was given placebos made of excipients and flavoring agents. Placebo formula: pyrosyrup (edible), apple green (edible), and lactose (medicinal). Placebos were packaged, shaped, and colored to be the same as JSD. Placebos were produced by the Placebo Experimental Center of School of Pharmacy, Chengdu University of Traditional Chinese Medicine, which had no efficacy or side effects. The external washing decoction was placed in a special vessel and air-dried to lukewarm for patients to take sitz bath for 30 min/time. Then, the bases of incisions were packed outward along with a gauze in moderate tightness. The incisions were covered with sterile gauze and fixed with adhesive plaster. The wound dressing was changed once a day for 4 weeks. We followed up the participants at the 8th, 12^th^, and 16th weeks after the dressing change was finished to record and analyze the recurrence.

### 2.9 Randomization and allocation concealment

Randomization was performed by an independent statistician (CZQ). Random sequences were generated by BMI SPSS Statistics 24.0 software. Given the number of seeds, 60 participants were randomly assigned to the intervention and placebo groups. The random distribution table was determined in triplicate, with one copy for the project leader (KJ), one for the pharmacist (TX), and another one for the statistician (CZQ).

### 2.10 Blinding

This study is designed to be a double-blind trial. During the experiment, neither participants nor researchers had group information. The medication number, label, and packaging for each participant were prepared from 001 to 030. Random numbers were sealed in double opaque envelopes, which were kept by CYQ, who was responsible for the blind method management. Every participant received their corresponding emergency letters, which were kept until the end of the trial.

### 2.11 Outcome measures

**FIGURE 3:**
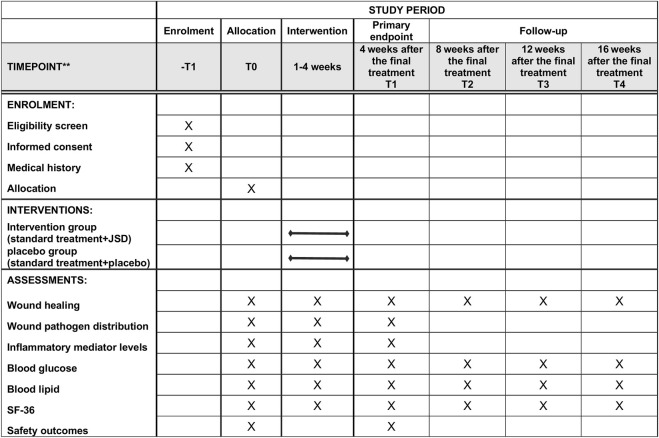
SPIRIT figure showing the time points for enrollment, interventions, and assessment.

Assessments were performed at the baseline (T0), 4 weeks after the final treatment (T1, primary endpoint), 8 weeks after the final treatment (T2, secondary endpoint, first follow-up 8 weeks after the final treatment), 12 weeks after the final treatment (T3, tertiary endpoint, second follow-up 12 weeks after the final treatment), and 16 weeks after the final treatment (T4, final endpoint, third follow-up 16 weeks after the final treatment). The timeframe of data collection and assessments is shown in Figure 3 (the SPIRIT figure).

### 2.12 Primary outcomes

#### 2.12.1 Wound healing

Assessments were at T0, T1, T2, T3, and T4. At the same time, weekly assessments were conducted during weeks 1–4 of intervention.

1) Wound healing rate: The wound area on the first postoperative day was the original area. The maximum length and width of the wound were measured with the calculated area. Formula: Wound healing rate = (original area-current area)/original area × 100%.
2) Wound healing time: From the start of the dressing change to the complete epithelialization and healing of the wound.
3) Wound secretion score (Table 2) (Gould et al., 2021): Wound with abundant secretion, two or more pieces of penetrated gauze, dressing change more than twice a day, 3 points; wound with much secretion, one piece of penetrated gauze, dressing change twice a day, 2 points; wound with little secretion, no penetrated gauze, 1 point; and wound with smooth surface and no obvious secretion, 0 point.
4) Wound edema score (Table 2) (Gould et al., 2021): Wound with severe edema which is significantly more than the wound edge, surgical resection needed, 3 points; wound with obvious edema which is more than the wound edge, need dressing change to remit, 2 points; wound with mild edema, 1 point; and wound with no edema, 0 point.
5) Granulation tissue color score (Table 2) (Gould et al., 2021): Bright red, 1 point; light red, 2 points; and purple, 3 points.
6) Anal function evaluation: The Wexner incontinence score was used to evaluate the anal function of participants after wound healing (Zhang et al., 2020a), including the ability of the anus to control the intestinal fluid, bowel gas, and loose and formed stools.
7) Anal pain score: The pain index tested before the dressing change on the first postoperative day was the baseline pain. The pain index after the first postoperative day was tested within 2 h after the dressing change. Pain values were tested using the visual analogue scale (Anal pain visual analogue scale, VAS) (Bondi et al., 2017).

**TABLE 2:** Scale for assessing wound healing.

Wound secretion score (Gould et al., 2021)	Score
Would with smooth surface and no obvious secretion	0
Would with little secretion and no penetrated gauze	1
Wound with much secretion, one piece of penetrated gauze, and dressing change twice a day	2
Wound with abundant secretion, two or more pieces of penetrated gauze, and dressing change more than twice a day	3
Wound edema score (Gould et al., 2021)	Score
Wound with no edema	0
Wound with mild edema	1
Wound with obvious edema, which is more than the wound edge and need a dressing change to remit	2
Wound with severe edema, which is significantly more than the wound edge, and surgical resection is needed	3
Granulation tissue color score (Gould et al., 2021)	Score
Bright red	1
Light red	2
Purple	3

#### 2.12.2 Distribution of pathogens on wound

Wound samples of participants were collected for pathogen isolation and cultured to perform identification on the automatic pathogenic microorganism identification instrument (Mark Biotechnology, United States). Weekly assessments were conducted during weeks 1–4 of intervention.

#### 2.12.3 Levels of inflammatory mediators

ELISA was used to measure the levels of IL-6 and TNF-α in related tissues of wound drainage collected from the participants. The streptavidin-peroxidase (S-P) method was used to perform immunohistochemical staining on wound edge tissue samples, which were observed under a 400-fold light microscope and semiquantitatively measured using a medical image analysis system. Weekly assessments were conducted during weeks 1–4 of intervention.

#### 2.12.4 Blood glucose

Blood glucose was measured four times per day by using a glucometer, including fasting and 2-h postprandial blood glucose of breakfast, lunch, and dinner.

### 2.13 Secondary outcomes

The assessments were at T0, T1, T2, T3, and T4. At the same time, weekly assessments were conducted during weeks 1–4 of intervention.

1. Blood lipids: total cholesterol (TC), total glycerides (TG), high-density lipoprotein (HDL), and low-density lipoprotein (LDL).
2. Quality of the life evaluation scale (Short-Form Health Survey 36, SF-36) (Wang et al., 2021).

### 2.14 Safety outcomes

The assessments were at T0 and T1.

1. Heart rate, blood pressure, temperature, and respiration.
2. Blood routine, stool routine, and urine routine.
3. Liver function: alanine aminotransferase, aspartate aminotransferase, γ-glutamyl transferase, alkaline phosphatase, and total bilirubin.
4. Renal function: blood urea nitrogen and creatinine.
5. Electrocardiogram.

### 2.15 Adverse events

Every adverse event (AE) was recorded, including the start date, end date, degree, relationship between the study drug and AE, and whether the participant is still involved in the study. Any serious adverse event (SAE) was reported to the Research Ethics Committee (REC) within 24 h. If any AE happened on any participant, the researcher asked the participant to stop using the washing decoction and determine if the event is related to the study drug formulation. If necessary, researchers took emergency safety measures to protect the participant from direct harm. If AE persisted, we followed it up until it was resolved. The AE in this study does not include complications clearly related to anal fistula, such as necrosis, prolonged healing, discharge, infection, pain, and itching.

### 2.16 Sample size

According to the sample size estimation method for the comparison of two sample means, we preset the sample size of the placebo group and the intervention group for them to be equal. The calculation formula is as follows:

$$n=2\sigma^{2}\times \frac{f\left(\alpha ,\beta \right)}{{\left(\mu_{1}-\mu_{2}\right)}^{2}},$$

where *n* represents the overall sample size of the study, μ1 and μ2 are the mean values of the control group and the test group, respectively, and σ is the standard deviation. We set that the probability of making type I error in this study shall not be greater than 5%, and the probability of making type II error at the same time shall not be greater than 10%, that is, α = 0.05 and β = 0.1. Referring to Chinese literature reports on TCM external washing for the treatment of postoperative wound of anal fistula, the wound healing rate (%) in literatures was set as the clinical efficacy observation, the mean value was 33.35, and the standard deviation was 2.29 in the intervention group, and the mean value was 27.33 and the standard deviation was 2.67 in the placebo group. The estimated loss of the follow-up rate was 20%, so the total study sample size was 60, of which 30 was for the intervention group and 30 was for the placebo group.

### 2.17 Data and sample collection

Three study members were trained to master the criteria and methods of case collection to minimize the selection bias. During data collection, the medication of participants was recorded in detail, and the interference and contamination factors in the statistical analysis were excluded.

On the consent form, the participants were asked if they agreed with the use of their data or they chose to withdraw from the trial. This trial does involve collecting biological specimens for storage. All samples were destructed after use.

### 2.18 Data management and monitoring

An independent Data and Safety Monitoring Board has been established to monitor the conduct and safety of the study. Two members of the research group (YH and XZ) entered the collected information into the Chinese Clinical Trial Management Public Platform under a confidential condition. The hard copy records were preserved at a locked office. No one will be able to change or use the hard copy and electronic data without the authorization of our group.

The investigators did their best to avoid missing clinical trial data. In the process of dealing with missing data, we chose different processing methods, according to the judgment of data managers on the mechanism of missing data. At the same time, it is necessary to make a sensitivity analysis of the test results.

### 2.19 Adherence to study interventions

During the study, we provided clear oral and written instructions to encourage and monitor the interventions. If necessary, further personalized guidance was provided. All unused medication was returned and recorded.

### 2.20 Data analysis

All data analyses were conducted by SPSS 24.0 software. Student’s t-test was performed on continuous normally distributed variables, the Wilcoxon rank-sum test on non-normal variables, and Pearson’s χ^2^ test on categorical variables. Data were presented as the mean ± standard deviation for continuous variables or percentage values for categorical variables. The statistical significance level was set at *p* < 0.05, and all the statistical tests were two-sided.

### 2.21 Frequency and plans for auditing trial conduct

The frequency and plans for the auditing trial conduct are as shown in Table 1. The auditing trial conduct was performed by a team independent from the investigators and the sponsor.

## 3 Discussion

Anal fistula is one of the common diseases in anorectal department, which is a tube located between the skin and rectum (Zheng et al., 2018; Garg, 2019). It is caused by chronic infection and epithelialization of the drains (Hokkanen et al., 2019; Sahnan et al., 2019). Diabetes mellitus is a risk factor for slow wound healing after anal fistula surgery (Wang et al., 2014; Mei et al., 2019), which can influence the development and outcome of patients with anal fistula (Mei et al., 2019). Surgery is currently the preferred treatment for anal fistula (Andreou et al., 2020; Mei et al., 2020; Wang et al., 2021). However, diabetic patients with large wounds after anal fistula surgery have an increased probability of wound infection, accompanied by pain, itching, swelling, and exudation, which are not easy to heal. Therefore, it is particularly important to shorten the wound healing time (Yang et al., 2017; Zhu et al., 2020).

As is known to all, TCM plays an important role in maintaining the health of Asians (Hao et al., 2017; Dai et al., 2020). TCM external washing has been widely used in clinical practice because of its reliable efficacy and fewer side effects (Guo et al., 2015; Zhang et al., 2020b). Despite the extensive clinical practice, the clinical literature on it for the treatment of postoperative wounds in diabetic patients with anal fistula is still not sufficient (Yang et al., 2017; Zhu et al., 2020). The clinical study we proposed will be the first randomized, double-blind, placebo-controlled, multi-center clinical trial study to assess the efficacy and safety of TCM external washing (JSD) in the treatment of postoperative wounds in diabetic patients with anal fistula.

However, the study still has some limitations due to the small sample size of the subjects. On the other hand, the action mechanism of JSD is still not clear and needs further investigation.

### 3.1 Trial status

The recruitment for the trial started in June 2022 and is expected to be completed in December 2022.

### 3.2 Modification of the protocol

Any changes in the program will be agreed upon by the project leader and supervisor. The sponsor and all members of the research team will be informed after approval by the ethics committee.

Data availability statement

The original contributions presented in the study are included in the article/supplementary material. Further inquiries can be directed to the corresponding author.

## Abbreviations

AE, adverse event; HDL, high-density lipoprotein; JSD, Jiedu Shengji decoction; LDL, low-density lipoprotein; REC, Research Ethics Committee; SAE, serious adverse event; SF-36, Short-Form Health Survey 36; SPIRIT, Standard Protocol Items: Recommendations for Interventional Trials; TC, total cholesterol; TCM, traditional Chinese medicine; TG, total glyceride.
