# Supplementary material for: Efficacy and safety of traditional Chinese medicine external washing in the treatment of postoperative wound of diabetes complicated with anal fistula: Study protocol of a randomized, double-blind, placebo-controlled, multi-center clinical trial
Source: Front Pharmacol. 2022 Dec 14;13:938270. doi: 10.3389/fphar.2022.938270 (PMC9795008; doi:10.3389/fphar.2022.938270)
Supplement: Supplementary file 2 [file Table2.DOCX]

**Informed Consent Form**

Name of participant: Gender: Age: Registry No:

Dear participant,

The purpose of this study is to provide high-quality evidence regarding the efficacy and safety of JSD in the treatment of postoperative wounds in diabetic patients with anal fistula and a new treatment strategy for clinicians and patients.

The study was designed to be a randomized, double-blind, placebo-controlled, multicenter clinical trial. If you agree to participate, you will receive the following tests to make sure that you are eligible to take part in the study:

The doctor will ask and record your medical history, clinical symptoms and signs, medications you are taking and took, and perform a physical examination.

If you meet the inclusion criteria, you will be randomly assigned to study group or control group and receive the treatment for 4 weeks and the follow-up for 3 months. You must come to the hospital at the appointment time agreed by the doctor and you. Your follow-up is very important because the doctor will determine whether the treatment you receive works and provide guidance in time. You must avoid other medications during the study. If you need any other treatment, please inform your doctor in advance.

The participation in this study is entirely voluntary. Participants may be withdrawn at any time during the study without affecting the relationship between you and researchers. There is no loss in economic aspect for you.

The research will strictly protect your privacy according to the principles of the Declaration of Helsinki. All information in this study will be kept confidential, and your private information will not appear in the research summary and published literature. This study has been ethically reviewed by the Medical Ethics Committee of Hospital of Chengdu University of Traditional Chinese Medicine (Ethical review document number:2022KL-018).

**Voluntary Subject Statement**

I have learned about the requirements of the clinical study in detail and the potential adverse reactions associated with it. I voluntarily participate in this clinical study, receive treatment on time, and fill out the relevant health questionnaire in accordance with the requirements of the research program. If adverse reactions occur, I will report to the doctor promptly. At the same time I know that the doctor will give positive response for my possible adverse reactions. I am also entitled to withdraw from the study at any time for any reason. However, if there are no special circumstances, I will cooperate with the doctor to complete the clinical study. My participation and the personal data in the trial are confidential. I agree with my doctor, the relevant regulatory authorities, and the ethics committee to review my information as required.

I (signature) relative (signature) (Relationship)

Date: D M Y

**Researcher Declaration**

I have fully elaborated the requirements of the observational study in detail and the potential risks or discomforts to the above participant/relative and answered their questions. To the best of my knowledge, the participant/relative has been informed adequately and has consented.

Researcher’s signature Date: D M Y

*In the event of inconsistency or discrepancy between the Chinese version and the English version, the Chinese language version shall prevail.*
